# Data on hepatic lipolysis, adipose triglyceride lipase, and hormone-sensitive lipase in fasted and non-fasted C57BL/6J female mice

**DOI:** 10.1016/j.dib.2016.03.033

**Published:** 2016-03-11

**Authors:** Phillip M. Marvyn, Emily B. Mardian, Ryan M. Bradley, Kristin A. Marks, Robin E. Duncan

**Affiliations:** University of Waterloo, Department of Kinesiology, Faculty of Applied Health Sciences, 200 University Avenue W., BMH1110, Waterloo, Ontario, Canada N2L 3G1

## Abstract

Liver homogenates produced from fasted and non-fasted C57BL/6J female mice were assayed for total lipolytic activity measured as hydrolysis of [9,10-^3^H(N)]-triolein into [^3^H] free fatty acids (FFA). Liver homogenates were also used for immunoblotting to determine levels of the lipolytic enzymes adipose-triglyceride lipase (ATGL) and hormone-sensitive lipase (HSL), as well as site specific phosphorylation at the 14-3-3 binding site of ATGL and the serine 565 and serine 660 sites of HSL. Significantly higher triolein hydrolysis activity was observed in fasted liver samples, as well as a significant increase in total ATGL and a significant decrease in HSL phosphorylation at the S565 site.

**Specifications table**

**Table:** 

Subject area	*Biology*
More specific subject area	*Lipid biology*
Type of data	*Graph*, *figure*
How data was acquired	*Radiochemical in vitro TAG hydrolase assay for measure of lipolytic activity and immunoblotting for protein expression*
Data format	*Radiochemical in vitro TAG hydrolase assay* (*analyzed*), *Immunoblots* (*raw and analyzed*)
Experimental factors	*Livers were harvested from C*57*BL*/6*J female mice fed a standardized diet provided ad libitum or following a* 16 h *fast*
Experimental features	*Liver lipolytic activity was determined by radiochemical in vitro hydrolase assay, lipolytic enzyme protein expression was determined by SDS-PAGE separation, immunoblotting and quantification by densitometry analysis*
Data source location	*University of Waterloo*, *Waterloo*, *Ontario*, *Canada*
Data accessibility	*Data is provided within the article*

**Value of the data**

• These data are of value to researchers interested in hepatic lipolysis regulation.
• These data are valuable to researchers interested in hepatic lipid metabolism in female mice.
• These data are valuable to researchers interested in the effects of fasting on the liver.

## 1 Data

**Fig. 1:**
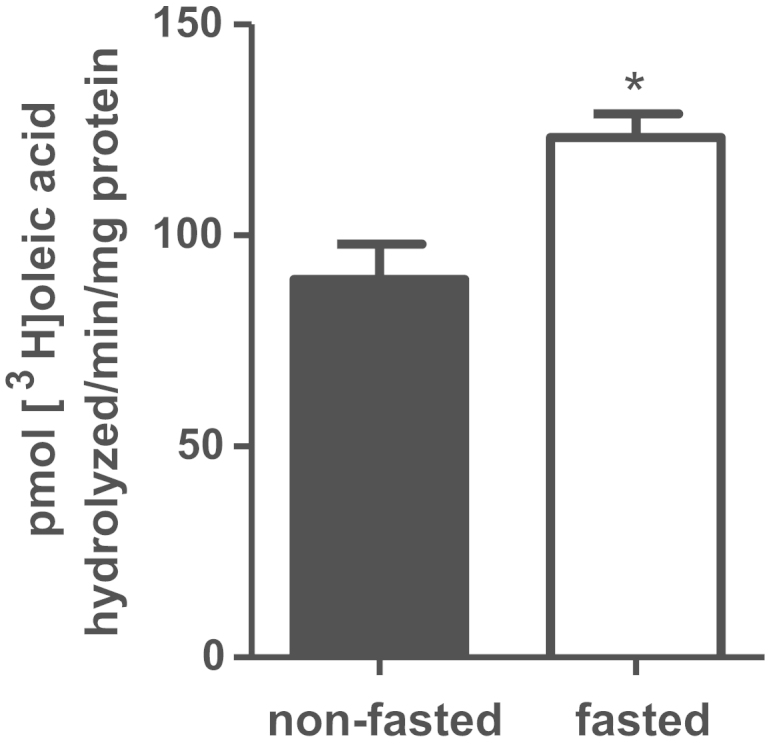
Data on [9,10-^3^H(N)]-triolein hydrolase activity of liver homogenates from fasted and non-fasted female mice. Liver homogenates were incubated with [^3^H]-triolein for 1 h, prior to extraction and resolution of total lipids by thin layer chromatography. The band corresponding to [^3^H]-free fatty acids was scraped and analyzed by scintillation counting. (*P<0.05) (*n*=5).

Studies have not yet reported in female mice the regulation of hepatic triolein hydrolase activity and changes in hepatic levels of total and phosphorylated adipose triglyceride lipase (ATGL) and hormone sensitive lipase (HSL) in response to short term fasting. Homogenates from fasted mice were assayed for *in vitro* [9,10-^3^H(N)]-triolein hydrolase activity and were found to have a 38% greater rate of release of radiolabelled FFA (123.2±5.66 pmol/min/mg protein) compared to homogenates from non-fasted mice (89.5±8.48 pmol/min/mg protein) (Fig. 1).

**Fig. 2:**
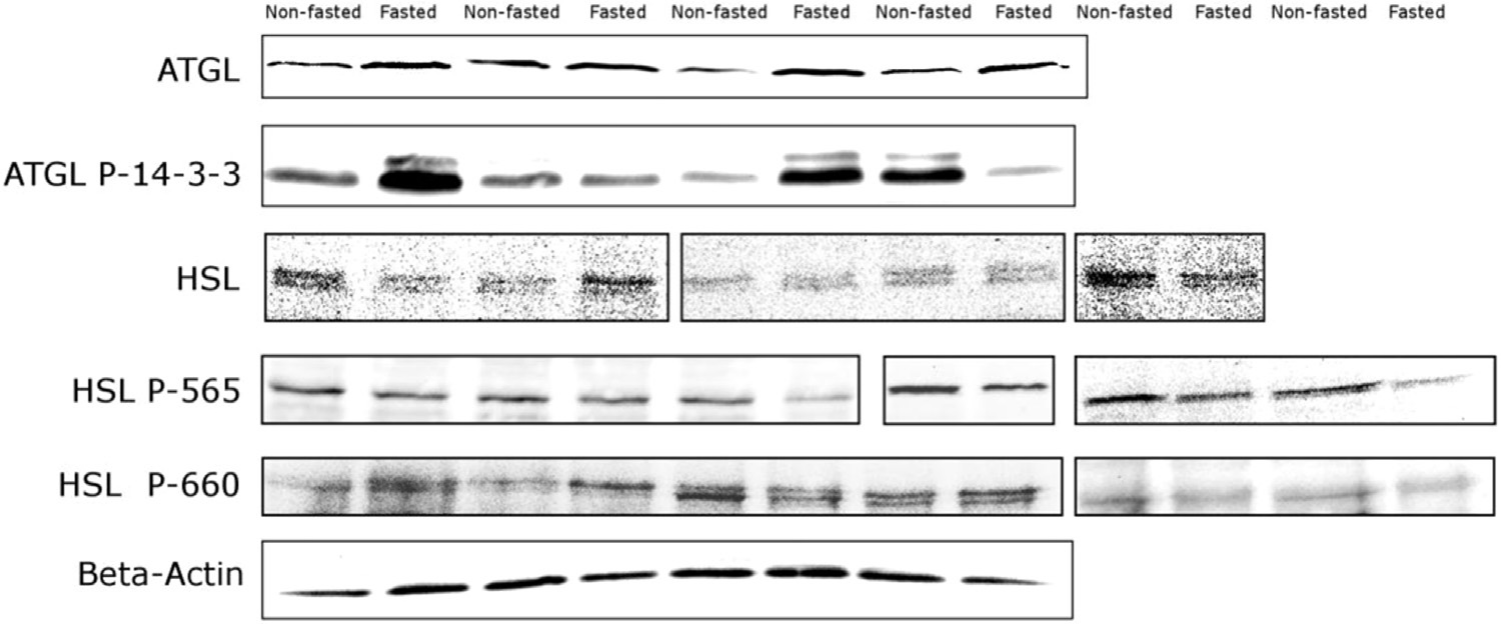
Data on the immunodetected levels and site-specific phosphorylation of ATGL and HSL in the livers of fasted and non-fasted mice. Immunoblots for lipolytic enzymes in the non-fasted and fasted mouse liver. Image borders represent independent immunoblots. Each well represents a different individual mouse.

**Fig. 3:**
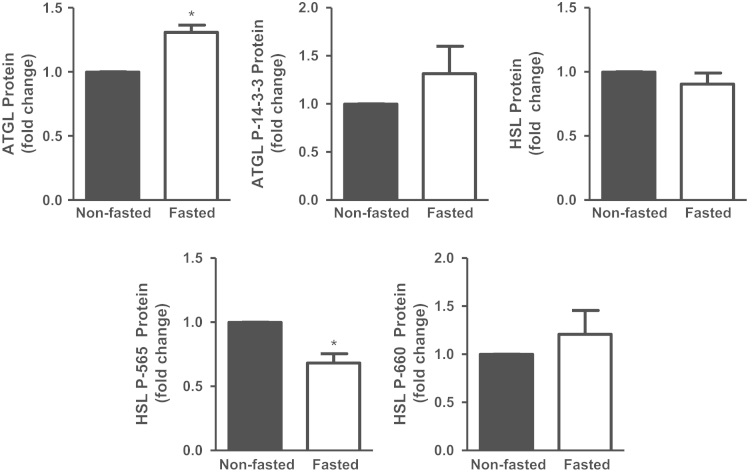
Data on the quantification of immunodetected levels of ATGL and HSL in the livers of fasted and non-fasted mice. Liver enzyme immunoblots were quantified by densitometry, depicted as fold change compared to non-fasted animals. (*P<0.05) (*n*=4–6).

The same liver homogenates were also subjected to SDS-PAGE separation and immunoblotting. These data demonstrate that total ATGL protein level was significantly (1.4 fold) more abundant in livers of fasted compared to non-fasted mice, but phosphorylation of ATGL within the 14-3-3 binding domain was not significantly increased in this data set (Fig. 2, Fig. 3). Total hepatic HSL protein levels were not significantly different between fasted and non-fasted mice. Probing site-specific phosphorylation of HSL revealed a significant 26% decrease in HSL phosphorylation at the serine 565 phosphorylation site, but no significant change in phosphorylation at the serine 660 site (Fig. 2, Fig. 3).

## 2 Experimental design, materials and methods

### 2.1 Experimental animals and protocol

C57BL/6J mice were bred in the central animal facilities at the University of Waterloo from founders originally purchased from Jackson Laboratories (Bar Harbor, Maine, USA). Fourteen to sixteen week old female mice were group housed in a temperature and humidity controlled environment with a standard 12 h light:12 h dark cycle. Standardized rodent diet (Research Diets #D12450H, New Brunswick, New Jersey, USA) and water were provided to the mice *ad libitum* for 9 days. Half of the mice were randomly chosen for a 16 h overnight fast from 5 pm to 9 am prior to cervical dislocation and tissue collection as we have previously described [1]. Protocols were approved by the Animal Care Committee at the University of Waterloo, with all procedures performed in accordance with the Canadian Council on Animal Care.

### 2.2 Protein extraction

As we have previously described for kidney [1], liver was collected from female C57BL/6J mice and homogenized on ice using a Polytron® homogenizer (VWR) in lysis buffer containing 50 mM Tris, pH 7.4, 1 mM EDTA, 5 mM sodium fluoride, 10 mM sodium orthovanadate, and 0.1 M sucrose, with protease inhibitor cocktail (1:100). Samples were centrifuged at 10,000×*g* for 10 min at 4 °C followed by sonication. Protein concentration was determined using a bicinchoninic acid assay.

### 2.3 Radiochemical in vitro TAG hydrolase assay

A radiochemical triolein hydrolase activity assay was performed, essentially as we have previously described [1], with minor modifications. Briefly, protein lysates containing 100 μg of protein in 100 μl of lysis buffer were added to 100 μl of a reaction mixture containing 300 μM triolein with [9,10-3H(N)]triolein (0.15 μCi per reaction) (Perkin Elmer Radiochemicals, Massachusetts, USA), 100 μM sodium taurocholate, 2% BSA (w/v), 1 mM DTT, 25 μM egg yolk lecithin, and 50 mM potassium phosphate (pH 7.2) to initiate the reaction. Reactions were quenched after 1 h by the addition of 0.25 ml 2:1 chloroform:methanol at 37 °C for extraction as described by Bligh and Dyer [2] where quenched reactions were next mixed with 0.25 ml of chloroform, vortexed, mixed with 0.25 ml of ddH_2_O, vortexed again and centrifuged at 1000×*g* for 5 min. The organic phase was removed, dried with nitrogen gas, and reconstituted in chloroform followed by application to a silica gel G plate with a solvent front containing hexane:diethyl ether:glacial acetic acid (80:20:2, v/v/v). Neutral lipids were separated to resolve FFA product from TAG substrate (and DAG and MAG) and the band corresponding to FFA, as identified by comparison to known standards, was scraped and quantified by liquid scintillation counting.

### 2.4 Immunoblotting

Immunoblotting was performed using aliquots of liver homogenates prepared for the triolein hydrolase assay, essentially as previously described [1], with minor modifications. Briefly, equal amounts of protein lysates were mixed with 6X protein loading dye and heated for 5 min at 95 °C. Samples were electrophoresed on a 12% SDS-PAGE gel at 120 V for 1 h and transferred onto nitrocellulose membranes at 0.35A for 90 min. Membranes were blocked in a 3% blocker (w/v) in TBST (50 mM Tris–HCl, pH 7.4, 150 mM NaCl, 0.1% Tween-20) solution for 1 h. The membranes were incubated overnight at 4 °C with a primary antibody (Cell signaling, Danvers, MA, USA) at 1:1000 in 2% blocker (w/v) TBST, against the protein of interest. Membranes were washed 3× with TBST and incubated for 1 h with HRP-conjugated secondary antibodies (Santa Cruz Biotechnology, Dallas, TX, USA) at 1:5000 in TBST with 2% blocker. Membranes were washed 3× with TBST and visualized by enhanced chemiluminescence. BSA was used as a blocker for phospho-specific antibodies and skim milk powder was used for all others. For detection of phosphorylation of 14-3-3 binding motif in ATGL, 1000 μg of lysates were first immunoprecipitated with rabbit anti-ATGL at 1:200 and captured with protein A/G agarose beads that were washed 3× with lysis buffer prior to separation and detection by SDS-PAGE.

### 2.5 Statistical analysis

The data are expressed as means±S.E.M. Statistically significant differences between two groups were assessed by Student׳s *t* test. Significance is accepted at *P*<0.05.
